# Pleiotropic Role and Bidirectional Immunomodulation of Innate Lymphoid Cells in Cancer

**DOI:** 10.3389/fimmu.2019.03111

**Published:** 2020-02-04

**Authors:** Zhengwen An, Fabian Flores-Borja, Sheeba Irshad, Jinhai Deng, Tony Ng

**Affiliations:** ^1^KCL Breast Cancer Now Research Unit, Guys Cancer Centre, King's College London, London, United Kingdom; ^2^Centre for Immunobiology and Regenerative Medicine, Barts and The London School of Medicine and Dentistry, Queen Mary University of London, London, United Kingdom; ^3^Richard Dimbleby Department of Cancer Research, Comprehensive Cancer Centre, Kings College London, London, United Kingdom; ^4^UCL Cancer Institute, University College London, London, United Kingdom

**Keywords:** ILCs, cancer, tumor immune microenvironment, immunosurveillance, heterogenity, plasticicity, immunothearpy

## Abstract

Innate lymphoid cells (ILCs) are largely tissue resident and respond rapidly toward the environmental signals from surrounding tissues and other immune cells. The pleiotropic function of ILCs in diverse contexts underpins its importance in the innate arm of immune system in human health and disease. ILCs derive from common lymphoid progenitors but lack adaptive antigen receptors and functionally act as the innate counterpart to T-cell subsets. The classification of different subtypes is based on their distinct transcription factor requirement for development as well as signature cytokines that they produce. The discovery and subsequent characterization of ILCs over the past decade have mainly focused on the regulation of inflammation, tissue remodeling, and homeostasis, whereas the understanding of the multiple roles and mechanisms of ILCs in cancer is still limited. Emerging evidence of the potent immunomodulatory properties of ILCs in early host defense signifies a major advance in the use of ILCs as promising targets in cancer immunotherapy. In this review, we will decipher the non-exclusive roles of ILCs associated with both protumor and antitumor activities. We will also dissect the heterogeneity, plasticity, genetic evidence, and dysregulation in different cancer contexts, providing a comprehensive understanding of the complexity and diversity. These will have implications for the therapeutic targeting in cancer.

## Introduction

**Figure 1:**
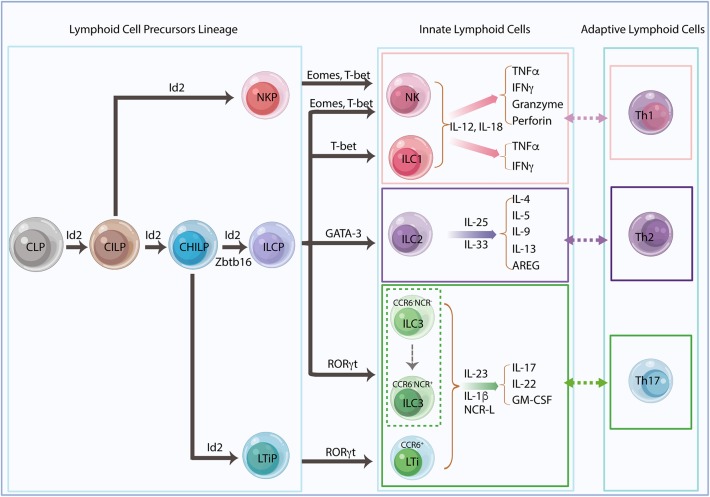
Innate lymphoid cell lineage and classification. Innate lymphoid cells (ILCs) derive from common lymphoid progenitors (CLPs) and give rise to common innate lymphoid progenitors (CILPs) and common helper innate lymphoid progenitors (CHILPs). CILPs differentiate into natural killer (NK) cell progenitors (NKPs) and then terminally differentiate into NK cells. CHILPs divide into innate lymphoid cell progenitors (ILCPs) and lymphoid tissue inducer progenitors (LTiPs), which generate all helper-like ILC subsets, ILC1s, ILC2s, ILC3s, and LTis, respectively. The transcriptional repressor Id2 is developmentally required and sequentially expressed in the ILC precursors. Id2-dependent precursors can further differentiate with lineage-specific transcription factors. Recent studies suggest that Zbtb16^+^ILCPs harbored extensive NK and/or ILC precursors potential, indicating a revised model for ILC differentiation. The different ILC subsets need their unique transcription factors for development and secretion of their signature cytokines in response to different stimulators, which mirror the phenotype and the function of adaptive helper T cells, Th1, Th2, and Th17, respectively.

Innate lymphoid cells (ILCs) derive from common lymphoid progenitors (CLPs) (1, 2) and are regulated by multiple endogenous signals including neuropeptides, hormones, cytokines and other alarmins (3). Tissue-resident ILCs represent a heterogeneous group of cells that interact with a wide variety of hematopoietic and non-hematopoietic cells through direct or indirect communication. These ILCs act as functional effectors contributing to the integration of innate and adaptive immune responses and the orchestration of physiological and pathological processes throughout the body (1). ILCs are considered to be counterparts of T-cell subsets resembling both their phenotypical and functional characteristics. Five subgroups of ILCs have been classified as natural killer (NK) cells and helper-like ILCs (ILC1s, ILC2s, and ILC3s) and lymphoid tissue inducer (LTi) cells in terms of their lineage-determining transcription factors and cytokine secretion profiles according to a redefined nomenclature (4) (Figure 1). NK cells derive from NK-cell precursors (NKP), which directly differentiate from common innate lymphoid progenitors (CILPs), whereas other ILCs derive from common helper innate lymphoid progenitors (CHILPs) (2, 5–7). CHILP can differentiate to ILC precursors (ILCPs) and LTi precursors (LTiPs) that give rise to all ILC subsets (ILC1, ILC2, and ILC3) and LTis, respectively (4, 8). The transcriptional repressor, inhibitor of DNA binding 2 (Id2), is sequentially expressed in the ILC lineage framework, and Id2-dependent precursors can further differentiate with lineage-specific transcription factors (9). Data from both humans and mice demonstrate that LTi cells and conventional NK (cNK) cells are developmentally related yet represent distinct lineages (10–12). Recent studies reveal the previously unappreciated ILCP heterogeneity by using polychromic reporter mice to identify the ILCP lineage. The results suggest that a fraction of CHILP highly expresses PLZF (Zbtb16) and PD-1 and retains potential for all CD127^hi^ILCs but not LTis. In addition, these cells can also generate NK cells that express Eomes and perforin, indicating additional lineage potential (13, 14). Likewise, an analysis of Id2 and PLZF reporter mice reveals that Id2^+^Zbtb16^+^ILCPs define multipotent NK and/or ILCPs and are associated with loss of LTi potential at a clonal level, suggesting a revised model for ILC differentiation that redefines the cell-fate potential of helper-ILC-restricted Zbtb16^+^ILCPs (15) (Figure 1).

NK cells and ILC1s share the same transcription factor T-bet and produce type 1 cytokines, including interferon-gamma (IFNγ) and the tumor necrosis factor-alpha (TNFα), in response to interleukin (IL)-12 and IL-18, whereas NK cells also require the transcription factor eomesodermin (Eomes) and produce cytotoxic proteins, perforin, and granzymes in mice and granulysin in humans (16). NK cells and ILC1s are functionally analogous to cytotoxic CD8^+^ T cells and CD4^+^ T helper1 (Th1) cells, respectively. ILC2s depend on GATA3 and produce IL-4, IL-5, IL-9, IL-13 (17, 18), and amphiregulin (AREG) (19) in response to IL-25, IL-33, and thymic stromal lymphopoietin (TSLP), which resembles the Th2 cells response (4). ILC3s are heterogeneous but consistently require transcription factor RORγt for their development and function. LTis are related to ILC3s with distinct functions in the secondary lymphoid organ formation during both embryonic and adult stages of development (8, 11, 20). In general, these cells found in adult mice are termed LTi-like cells, which have a similar phenotype to LTis (21). Both ILC3s and LTis are capable of producing IL-17, IL-22 and granulocyte-macrophage colony-stimulating factor (GM-CSF) in response to IL-23, IL-1β, or natural cytotoxicity receptor ligands (NCR-L) that reflect Th17 response (3, 22). Regardless of the functional association with ILC3s, LTis are considered a separate ILC lineage. Fetal liver-derived LTiPs express α4β7 integrin and C-C Motif Chemokine Receptor 6 (CCR6), whereas adult LTis derived from bone marrow precursors display upregulated RORγt in a Notch-dependent manner (9, 20). Adult mice also have T-bet-expressing CCR6^−^NKp46^+^ILC3s derived from CCR6^−^NKp46^−^ILC3s (23) (Figure 1).

In contrast to the polarized T-cell subsets that take over several days for activation and clonal expansion, most ILCs can produce significant amounts of cytokines upon stimulation without further differentiation. Hence, ILCs are considered to be the first line of defense to confront and sense the changes in the local environment and then react rapidly as the host response in peripheral tissue (24). Dysregulation of ILCs manifested by the changes in cell numbers or subset proportions is associated with diverse inflammatory diseases and cancer (25–28). The role of ILCs in cancer is ambivalent based on both protumor and antitumorigenic activities depending on their phenotype, the variety of cancer, and the tumor microenvironment (TME) context. The remarkable heterogeneity, distinct signature cytokines they produce, the various surface markers they express, and the plasticity among all the different subsets make ILC functions divergent and less comprehensible.

Given that the role of NK cells in cancer has been extensively reviewed (29, 30), this review will mainly focus on helper-like ILCs (ILC1s, ILC2s, and ILC3s) and LTis in the context of cancer. The plasticity of ILCs and their interactions with the extracellular matrix (ECM) and adaptive immune cells shape the TME. In return, changes in the secretion of cytokines by these cells also polarize ILC functions and influence plasticity under pathological conditions in the TME. These bidirectional interactions are crucial for controlling tumor growth and metastasis. Untangling the pleotropic roles and bidirectional regulations of ILCs in cancer will ultimately help to provide a rationale for the design of therapeutic strategies for cancer treatment.

## Innate Lymphoid Cells and Tumor Immune Microenvironments

Tumor-infiltrating leukocytes were first discovered in the 1800s, suggesting a functional relationship between immune cells and cancer (31). These infiltrating immune cells had been considered to have antitumor properties only until the last three decades, when the adverse role of promoting tumor progression has come into light. Tumor development and progression are profoundly influenced by a variety of resident host cells, stromal cells, the ECM, the blood and lymphatic vascular networks, infiltrating immune cells, and signaling molecules including growth factors, cytokines, and chemokines, collectively known as the TME (32–35). Recognizing the importance of the TME in cancer has revolutionized cancer treatment from targeting tumor cells to deciphering the tumor ecosystem complexity. This fundamentally determines the fate of the primary tumors whether it is eradicated or a premetastatic niche is established that favors metastatic dissemination (36, 37). Substantial immune cell infiltration varying in size, composition and distribution is a distinct characteristic present in almost all types of malignant tumors. These infiltrating immune cells, including tumor-associated macrophages (TAMs), mast cells, T cells and ILCs within the TME, comprise the tumor immune microenvironment (TIME) (26, 38–43).

Each of these immune components is believed to be involved in tumorigenesis, tumor invasion, and metastasis (44, 45). From immunosurveillance to tumor escape, infiltrating immune cells mutually interact with other cells within the TME niche and play a dual role that can either promote or attenuate malignant outgrowths. Given that tumor-induced immunological changes affect metastatic progression even before disseminated tumor cells reach secondary organs (38), targeting the premetastatic TIME is believed to be critical for the success of therapeutic strategies (40, 42, 46).

ILCs are tissue-resident cells in lymphoid and non-lymphoid organs and functionally act as sentinels to maintain and shape tissue microenvironment. They respond rapidly to environmental changes by secreting significant amounts of cytokines and expanding locally under physiologic and pathological conditions, suggesting a role for these cells in the early phases of tumorigenesis and in shaping the TME (46).

### ILCs in Extracellular Matrix Remodeling

ECM is a major component of the local TME, and its remodeling plays a prominent role in tumor development. ECM dysregulation promotes cancer cell invasion, induces angiogenesis and facilitates immune cell infiltration (47). Activated immune cells including ILCs closely interact with ECM, which may decisively affect the outcome of tumor progression (42).

**Figure 2:**
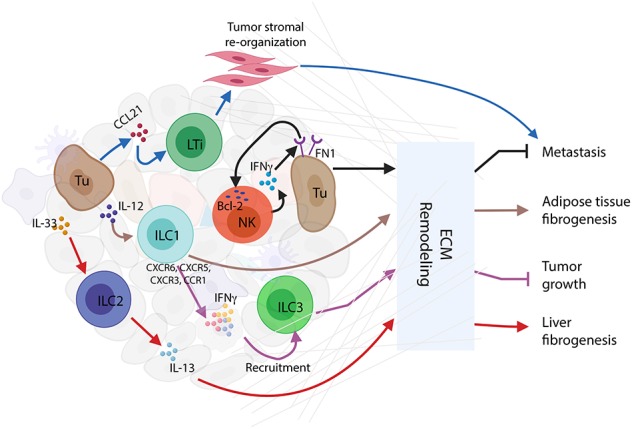
Innate lymphoid cells (ILCs) and extracellular matrix (ECM) remodeling. Activated ILC interactions with ECM in tumor immune microenvironment (TIME) conclusively affect the consequences of tumor progression. Interferon-gamma (IFNγ) secreted by natural killer (NK) cells stimulate tumor cells to express FN1 that causes ECM remodeling resulting in the inhibition of tumor metastasis. Mutually, FN1 stabilizes NK cell survival and facilitates NK cell migration through the induction of B-cell leukemia 2 (Bcl-2) expression. In response to IL-12, ILC1s upregulate several cytokines and chemokines, such as IFNγ, CXCR6, CCR5, CXCR3, and CCR1, to recruit ILC3s to the tumor site, thus causing ECM remodeling and tumor growth repression. Through ECM remodeling, adipose ILC1s contribute to adipose tissue fibrogenesis. Similarly, in mouse liver, IL-33-activated ILC2s release IL-13 to stimulate hepatic stellate cells to produce ECM proteins, thus inducing liver fibrogenesis. Moreover, tumor cells recruit CCL21 to activate LTis, whereby causing tumor stromal reorganization and facilitation of lymph node metastasis.

Among the ILC family members, NK cells were first identified as being involved in the remodeling of the tumor ECM. NKp46-mediated IFNγ secretion increases the expression of fibronectin 1 (FN1) by tumor cells leading to ECM structural changes in the primary tumors and decreased metastasis (48). Reciprocally, FN1 stabilizes NK cell survival and facilitates NK cell migration by inducing anti-apoptotic protein B-cell leukemia 2 (Bcl-2) (49). These data suggest a bidirectional regulation between NK cells and ECM remodeling. IL-12-responding ILC1s induce a signature TME characterized by strong upregulation of IFNγ and type I-associated chemokine receptors (CXCR6, CCR5, CXCR3, and CCR1) to recruit tumor invading *Rorc*^*fm*+^ILCs in a mouse model of melanoma (50). Adipose ILC1s were also found to contribute to adipose tissue fibrogenesis in humans (51). Other studies of ILC2s show that activation by IL-33 induces IL-13 secretion, which in turn stimulates hepatic stellate cells to produce ECM proteins leading to pathologic tissue remodeling in mouse liver (52) (Figure 2). Depletion of RORγt-dependent LTis and RORγt-independent ILCs impairs lung function and tissue repair, whereas IL-33/IL-33R signaling is critical for lung ILC response through the production of AREG via IL-13/ IL-22-independent mechanisms (53).

As described above, the remodeling of ECM causes organ dysfunction and contributes to chronic disease mechanisms including cancer. Innate immune cytokines derived from ILCs subsets directly regulate fibroblast functions that are independent of adaptive immunity (54). Taken together, these studies demonstrate a critical role for ILCs as important regulators of cytokine networks involved in ECM remodeling and the consequences for health and disease. This also suggests the clinical relevance of controlling pathologic remodeling to prevent metastasis.

### ILCs in Tumor Angiogenesis and Lymphatic Vascular Networks

Tumoral angiogenesis is a characteristic feature of tumor survival and progression, which differs considerably from the vascular structure generated for the regular blood vessels (55, 56). The formation of new blood vessels in tumors is driven by hypoxic tumor cells, tumor-associated stromal cells (TASCs), and the ECM in which they are embedded, producing vascular endothelial growth factor A (VEGFA) to initiate tumor angiogenesis (57). Tumor-infiltrating immune cells, such as ILCs, are also important pro-angiogenic mediators to increase VEGFA bio-availability and signaling during the angiogenic switch (58). ILCs stimulate endothelial cell proliferation and upregulation of adhesion molecules by releasing pro-angiogenic factors, contributing to immune cell recruitment and perpetuation of inflammation (42, 55, 56, 59).

**Figure 3:**
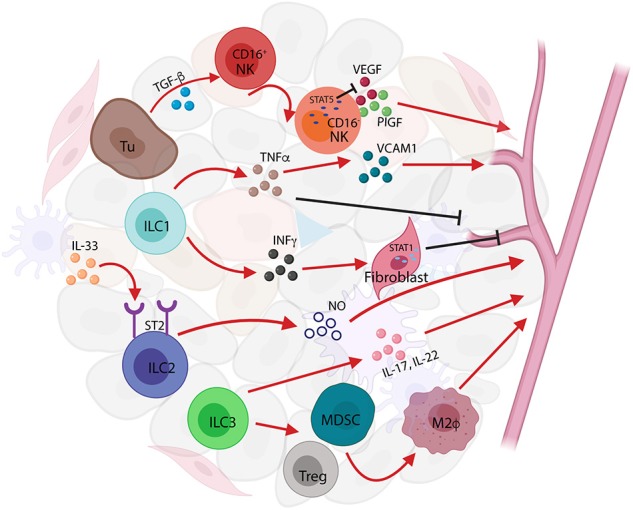
Innate lymphoid cells (ILCs) in tumor angiogenesis. ILCs act as tumor angiogenesis modulators by releasing pro-angiogenic factors and by inducing the recruitment and infiltration of immune cells to affect tumor-related inflammation. Transforming growth factor-beta (TGF-β) secreted by tumor cells activate natural killer (NK) cell to produce vascular endothelial growth factor (VEGF) and placenta growth factor (PIG) to induce tumor angiogenesis; conversely, the transcription factor STAT5 represses the expression of VEGF resulting in the inhibition of angiogenesis and tumor growth. ILC1s produce two signature cytokines, interferon-gamma (IFNγ) and tumor necrosis factor-alpha (TNFα), that are associated with cell proliferation and angiogenesis. TNFα secreted by ILC1s increases vascular cell adhesion molecule (VCAM)1 expression causing tumor vascular formation, whereas in a different context, TNFα-producing ILC1s can either destroy tumor vasculature or induce apoptosis acting as antitumor effectors. Furthermore, IFNγ released from ILC1s causes STAT1 activation, thereby inhibiting angiogenesis formation. ILC2s respond to IL-33 and induce angiogenesis and vascular permeability through ST2 receptor binding. IL-17 and IL-22 released by ILC3s promote angiogenesis via stimulation of vascular endothelia cell migration and cord formation. The indirect role of ILC3s in tumor angiogenesis is also shown in the recruitment of myeloid-derived suppressor cells (MDSCs), regulatory T cell (Treg) cells, and the promotion of M2-like macrophages in the tumor immune microenvironment (TIME).

Tumor-infiltrating NK cells, transformed from CD16^+^NK to CD16^−^NK subset upon transforming growth factor-beta (TGF-β) stimulation, predominate in non-small-cell lung carcinoma (NSCLC) and produce elevated levels of pro-angiogenic factors such as VEGF and placenta growth factor (PIGF) to sustain tumor progression (55, 60, 61). This pro-angiogenic effect can be reversed by transcriptional factor STAT5 via repressing VEGFA transcription in NK cells, in both mice and humans (62). The secretion of VEGF by NK cells is associated with angiogenesis in human melanoma, and breast and colon carcinoma (42). These studies suggest that the TIME is able to functionally affect NK cells by inhibiting their cytotoxic ability or promoting pro-angiogenic phenotypes by a wide array of cytokines and soluble factors such as TGF-β, PGE2, VEGF, and adenosine (55). Likewise, tumor-infiltrating ILC1s in mouse fibrosarcoma also showed upregulation of angiogenesis gene sets that also expressed in NK cells (63). ILC1s impaired tumor neovascularization mediated by IL-12, which required the presence of NK cells to induce endothelial cell cytotoxicity in lymphomas (64). ILC1s produce two signature cytokines, IFNγ and TNFα, which are associated with cell proliferation and angiogenesis. IFNγ activates the transcription factor STAT1 to inhibit tumor cell proliferation and angiogenesis (65, 66). TNFα can either destroy tumor vasculature and induce apoptosis as an antitumor effector or stimulate the expression of angiogenic and growth factors to promote tumor formation and growth (38). ILC2s in response to IL-33, the cytokine that induces angiogenesis and vascular permeability through ST2 receptor binding (67), can enhance the re-epithelialization and promote the restoration of skin integrity after injury (68). ILC3s contribute to preserving epithelial integrity and maintaining tissue homeostasis by the release of IL-22 (59). Secretion of IL-17 by ILC3s promotes angiogenesis via stimulation of vascular endothelial cell migration and cord formation, resembling those indirect angiogenic stimulators such as TGF-β and platelet-derived growth factor B subunit homodimer (PDGF BB) *in vivo* (69). The indirect role of ILC3s in tumor angiogenesis is also manifested by their recruitment of myeloid-derived suppressor cells (MDSCs) and regulatory T cell (Treg) cells, which in turn promote M2-like macrophages in the TIME (70, 71). Apart from IL-17 and IL-22, the LTi-like neuropilin (NRP)1^+^ILC3 subset was also found to release CSF2, TNFα, B-cell-activating factor, and CXCL8, in association with VEGF production that might contribute to angiogenesis (59) (Figure 3).

The other prominent feature of tumor angiogenesis is the expression of adhesion molecules such as vascular cell adhesion molecule (VCAM) and intercellular adhesion molecule (ICAM), which conveys the apparent tumor-immune privilege. In a subcutaneous melanoma mouse model, NKp46^+^LTi cells alter the tumor microvasculature upon IL-12 stimulation, which leads to upregulation of VCAM and tumor suppression (72). Indeed, LTis modulate not only blood vasculature but also the lymphatic vascular system. LTis induce mesenchymal stem cells (MSCs) to produce chemokines, CCL19, CCL21, or CXCL13, which promote lymphocyte recruitment and spatial compartmentalization (73). This cross talk also plays a role in promoting lymph node metastasis in breast cancer. In the 4T1.2 triple-negative breast cancer (TNBC) mouse model, ILC3s are recruited to the primary tumors by CCL21 and stimulate tumor stromal cells to release CXCL13, leading to enhanced tumor cell motility, lymphangiogenesis, and lymph node invasion by tumor cells (74). These data suggest that the number of infiltrating ILCs within the primary breast tumors could be used as a predictor of metastatic and malignancy potential (74).

Tumor angiogenesis and lymphatic vascular formation prompt tumor invasion and metastasis, the landmark events that transform a locally growing tumor into a systemic metastatic and life-threatening disease. As tumor-infiltrating ILCs can polarize the TME to either protumor or antitumor effects by the modulation of angiogenic activities and lymphatic vascular networks, these cells represent valid targets for antitumor immunotherapy and cancer preventive strategies (55).

### Interplay Between ILCs and Cytokines, Chemokines and Growth Factors in Tumor Immune Microenvironment

Initiation of ILC response relies on sensing the cytokines, alarmins, and inflammatory mediators that are derived from tissue sentinels such as myeloid cells, dendritic cells (DCs) and macrophages, or epithelial cells to translate environmental signals into a specific cytokine profile (75). The complex, diverse and dynamic interplay with surrounding environments amplifies ILC signaling and determines their function. Tumor-infiltrating immune cells engage in an extensive and dynamic interaction with TIME and shape the TME, whereas tumor cells also induce an immunosuppressive microenvironment by the secretion of the cytokines and other soluble factors (33).

In a model of subcutaneous melanoma, ILC1s respond to IL-12, produced by tissue sentinels such as DCs and macrophages, and alter the TME at an early stage of tumor development to facilitate tumor suppression by infiltrating immune cells (72). The production of TGF-β in the TME drives ILC fate outcomes between the ILC subsets from NCR^−^ILC3s to NCR^+^ILC3s (76). In human ovarian cancer, IL-22-producing ILC3s prevent tumor-infiltrating lymphocyte activation and proliferation (27), whereas the release of IL-22 and IL-17 by ILC3s modulates intestinal immune pathology (77). In a preclinical mouse model of human breast cancer, RORγt^+^ILC3 promoted lymphatic metastasis by modulating the local chemokine milieu in the TME (74). Genetic mapping of the fate of ILC3 revealed that different subsets of ILC3 are phenotypically adapted to the local tissue environment they invade, indicating that the function of ILCs is shaped not only during their lineage commitment but also by the TME (50). Although ILCs are characterized by the distinct signature cytokine they express, tissue-derived cytokines can extrinsically shape ILC function (50), and this cross talk can drive both positive and negative consequences in tumor progression (78).

Interestingly, exosomes (extracellular vesicles, 50–150 nm in size released from the endocytic compartment) (79) derived from both tumors and stromal cells have been implicated as signaling mediators between cancer cells and surrounding cells that comprise the TME and contribute to therapy resistance. Several studies have identified the role of exosomes in premetastatic niche establishment that is favorable for future dissemination and metastatic seeding through the communication with immune cells like NK cells, macrophages, B cells, and T cells. Recent studies also revealed that chemotherapy-elicited extracellular vesicles in breast cancer cells can promote premetastatic niche formation in lung by delivering annexin 6 to induce CCL2, which promotes monocyte activation (80). The direct correlation of exosomes with ILCs has not been explored yet, although AREG, which is produced by ILC2s, can be found in distinct nanoparticles (DNPs), a subset of extracellular vesicles that are smaller than 50 nm with a dot-shaped morphology (81). Further investigation of ILCs and exosome communication would potentially allow the development of effective clinical applications that may overcome the therapeutic resistance.

### Interplay Between ILCs and Adaptive Immunity

Emerging evidence indicates that ILCs play an important role in coordinating tissue-specific adaptive immunity and act as master regulators in homeostasis and diseases. Substantial studies have demonstrated the direct communication between ILC2s and ILC3s with the adaptive immune system, whereas with ILC1s, it remains less well-investigated (21).

**Figure 4:**
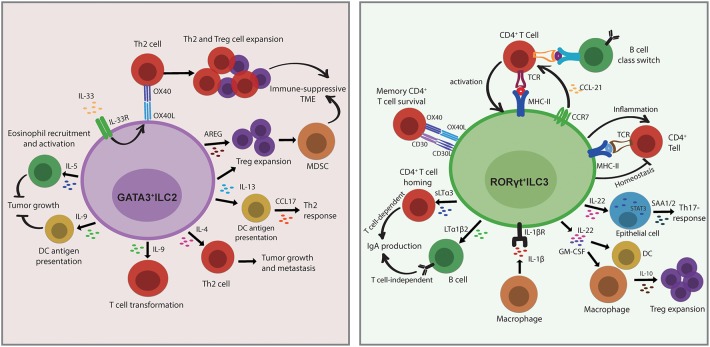
Innate lymphoid cells (ILCs) modulate adaptive immunity by direct or indirect interactions with other cells in the tissue. ILC2s functionally resemble Th2 cells and play a critical role in the type 2 immune response. The role of ILC2s in the establishment of an by direct interactions with Th2 cells through OX40L/OX40 under IL-33 stimulation or through indirect interactions by the section of IL-13 or amphiregulin (AREG), which then causes regulatory T cell (Treg) cell expansion and myeloid-derived suppressor cell (MDSC) activation. ILC2-derived IL-13 also stimulates dendritic cell (DC) recruitment and CCL17 production to provoke a Th2 response. ILC2s produce IL-4 to regulate Th2 differentiation, which may suggest a potential role in promoting tumor growth and metastasis. In terms of antitumor function, the release of IL-5 and IL-9 by ILC2s restrains tumor growth through eosinophils recruitment and activation and DC antigen presentation. Conversely, high expression of IL-9 is associated with T-cell transformation in humans, indicating that the anti-apoptotic properties on transformed cells promote tumorigenesis. ILC3s are important in the Th17 immune response. The direct interactions with CD4^+^T cells are either through MHC II/TCR or through OX40/OX40 and CD30L/CD30 to regulate T-cell survival or B-cell class switch, respectively. ILC3 migrate to the draining mesenteric lymph node to initiate an adaptive response that requires CCR7 expression. ILC3s promote IgA production in a T cell-dependent manner via the regulation of CD4^+^T-cell homing by sLTα3 secretion and also in a T cell-independent manner via control of B-cell homing by the release of LTα1β2. ILC3-derived IL-22 triggers epithelial serum amyloid A protein (SAA) production to induce local Th17 response in a STAT3-dependent manner. The secretion of IL-22 and GM-CSF by ILC3 stimulates macrophages and DCs to release IL-10, and this consequently results in regulatory T cell (Treg) cell expansion. In addition, IL-1β produced by macrophages activates ILC3 and leads to CD4^+^T-cell priming, whereas T cell-derived cytokines also cross-regulate ILC response, indicating the bidirectional interactions between ILC3 and T cell-mediated immunity.

ILCs share several developmental and transcriptional signatures with T-cell subsets but have additional and/or unique, non-redundant functions in T-cell polarization and effector functions by secretion of regulatory cytokines, antigen presentation, or direct cellular interactions within complex tissue microenvironments. ILC2s functionally resemble Th2 cells and play a critical role in the type II immune response. ILC2-derived cytokine IL-13 stimulates tissue DC recruitment to the draining lymph node for eliciting production of Th2 cell-attracting chemokine CCL17, causing the Th2 response (82). The protumor activity of IL-13 is also involved in the activation and differentiation of MDSCs by induction of Treg cell expansion to establish an immune-suppressive TME (83). These studies may indicate that the role of ILC2/IL-13 axis in the initiation of cancers is linked to extensive tissue remodeling. During intestinal or lung inflammation, tissue-specific IL-33 secretion upregulates OX40L expression on ILC2s resulting in protective Th2 immunity and Treg cell expansion, which could be related to the induction of a tolerant microenvironment and inhibition of antitumor responses (84). In addition, ILC2-derived AREG limits antitumor immunity by stimulating Treg cell expansion, supporting the establishment of an immune-suppressive TME (85). ILC2s have been suggested as a key source of Th2 master regulator IL-4 (86), and the previously unrecognized role of ILC2-derived IL-4 in Th2 differentiation may indicate the potential involvement of ILC2/T-cell interactions in promoting tumor growth and metastasis (38). Notwithstanding the protumor role of ILC2s in cancer, ILC2-derived cytokines such as IL-5 are involved in an antitumor function. In a melanoma model, increased IL-5 production by ILC2 resulted in the recruitment and activation of eosinophils, which was correlated with lung tumor metastasis. Moreover, ILC2-derived IL-9 has recently gained attention in the context of tumor biology and has functional importance as growth factor for ILC2 and survival factor for other cell types (83). In a melanoma model, IL-9 deficiency promoted tumor growth, whereas IL-9 treatment decreased metastasis (87). It is possible that the role of IL-9 in cancer is associated with the promotion of infiltrating DC antigen presentation (88). Conversely, high expression of IL-9 was associated with T-cell transformation in humans, indicating that the anti-apoptotic properties on transformed cells promote tumorigenesis (89) (Figure 4).

Increasing evidence over the past decade also indicated the role of ILC3s in bridging innate and adaptive immune responses. In a bone marrow chimeric mouse model, RORγt^+^ILCs (LTis) express OX40L and CD30L to regulate memory CD4^+^ T-cell survival but not that of CD8^+^ T cells in the homeostatic state (90), whereas in normal intestines ILC3s limit T-cell activation but decrease the regulatory effects under an inflammatory microenvironment through MCH-II presentation (91), indicating that the function of ILCs can be altered by mutual interaction with the microenvironment. ILC3s localize at the boundary between B-cell follicles and T-cell zone within intestinal draining lymph nodes to regulate T follicular helper cells (Tfh) responses and B-cell class switching via antigen presentation (92). In contrast to peripheral lymph nodes, resident ILC3s at mucosal draining lymph nodes express MHC-II and contribute to a distinct microenvironment constraining CD4^+^ T-cell response to commensal bacteria in a contact-dependent manner dependent on CCR7 (93). Commensal microbiota and IL-1β stimulate the release of GM-CSF and IL-22 by ILC3 through MHC-II-dependent interactions to promote intestinal Treg cell expansion (94, 95). In addition, IL-1β stimulation leads to activation of peripheral ILC3s and consequently primes the CD4^+^ T-cell immune response, whereas T cell-derived effector cytokines also cross-regulate the ILC response. The cognate interaction of ILC3 and CD4^+^ T cells is bidirectional, indicating an unappreciated role of ILC3s in T cell-mediated immunity (96). ILC3 also activates local and systemic inflammatory T cells by increasing TNF-like ligand 1A (TL1A) and IL-1β (82, 95). In the peritoneal B-cell compartment, RORγt^+^ILCs produce soluble lymphotoxin α3 (sLTα3) and membrane-bound lymphotoxin β (LTβ), both trimeric cytokines of the TNF superfamily, resulting in T cell-dependent and T cell-independent IgA production, respectively (97). Moreover, the adherent microbiota stimulates ILC3 to secrete IL-22 that triggers epithelial serum amyloid A protein (SAA) production to promote local Th17 responses in a STAT3-dependent manner (98) (Figure 4). The proliferative and anti-apoptotic capacities of ILC3-derived IL-22 may support malignant transformation in chronic inflammatory diseases. Those studies revealed the potential function of ILC3s as antigen-presenting cells and directly modulate adaptive immune responses. Even though ILCs show striking similarities to T-cell subsets, the non-redundant functions go beyond those shared with T cells through indirect and direct interaction with adaptive immune cells. In turn, adaptative immunity reciprocally regulates ILCs, which indicates that these bidirectional regulations are a crucial determinant of immune response within tissues. Given that the abundant evidence is mostly established under inflammatory conditions, a functional analysis between ILC3s and adaptive immunity in chronic inflammatory diseases may also imply the role of these interactions in the malignant transformations.

Collectively, these studies demonstrate key innate mechanisms in the regulation of the adaptive immune response and shed light on the tissue ILC-mediated control of adaptive immunity in orchestrating inflammatory and restorative tissue responses (99). Delineating the complex network of ILCs with other immune cells interactions will allow the manipulation of their specific function and ultimately open a way to innovative therapeutic strategies (41).

## Innate Lymphoid Cells and Cancer-Related Inflammation

Inflammation is a well-known hallmark of cancer that sustains cancer progression by providing growth factors, proangiogenic factors, and ECM modifying enzymes. During the different stages of tumor development, the inflammatory response plays decisive roles in tumor initiation, promotion, malignant conversion, invasion, and metastasis (100). Inflammatory conditions can be present before or after a malignant change occurs, which induces an inflammatory microenvironment to promote tumor development (101). Cancer-related inflammation in the presence of inflammatory cells and their mediators, such as cytokines, chemokines and prostaglandins, in tumor tissue is similar to that observed in a chronic inflammatory response (101). The character of the inflammatory response and the composition of the immune cell infiltration are indispensable to shape the TIME (33).

ILCs are believed to be resident, whereas NK cells recirculate. Given their distribution throughout the body, ILCs play a critical role in coordinating inflammation and cancer (75). ILC1s are highly abundant in the small intestine, liver, uterus, and salivary glands; ILC2s are enriched in the skin and lung; and ILC3s are abundant in the colon and tonsils (27, 102). Mucosal ILC1s are important in barrier defense through escalating Th1-like responses against pathogenic infections (99). During inflammation, a higher frequency of IFNγ-producing ILC1 subsets in mucosal tissue is positively correlated with patients having Crohn's disease compared with the normal tissue, indicating a role for ILC1 in the pathogenesis of gut mucosal inflammation (103). ILC2s secrete the type 2 cytokines IL-5 and IL-13 to control helminths, restrain parasitic infections (99), and drive airway hyper-reactivity in mouse models of allergic asthma (103, 104). Tissue-derived cytokines can also shape ILC function in inflammation, and this is largely achieved by the IL-22 pathway (78). In response to IL-23 stimulation, IL-22-producing ILC3s facilitate tissue protection from the acute phase of *Citrobacter rodentium*-induced colitis (105). ILC3-dependent protection also occurs at other mucosal sites, including the lung and oropharyngeal barrier through IL-17 and IL-22 (106–108). ILCs are important in tissue repair and remodeling after infection. IL-22^+^ILC3s limit inflammatory colitis, and their frequency correlates with mucosal repair in inflammatory bowel disease (IBD) (99). LTis are involved in tissue repair after clearance of lymphocytic choriomeningitis virus infection (109), and ILC2s assist lung tissue repair after influenza virus infection (53).

Indeed, ILCs act as a two-edged sword in inflammation. The same factors that inhibit acute inflammation and promote tissue repair can have pathogenic effects during chronic inflammation activation. IL-5-producing ILC2s boost lung inflammation by recruiting eosinophils that are the main allergic effectors, whereas maintenance of IL-13 production stimulates collagen deposition leading to chronic fibrotic tissue damage and pulmonary fibrosis in hyper-reactive lung airways (110–112). In the colon, chronic infection-induced IL-23 triggers RORγt^+^ILC accumulation and increases IL-17 and IFNγ production that results in the bacteria-driven colitis (77). Furthermore, the protumor activities of ILC3 are mainly associated with chronic inflammation by IL-23-induced secretion of IL-17 and IL-22 (113). Antigen-presenting ILC3s act as a checkpoint to control Tfh and B cells response toward mucosal-dwelling microbiota (92). Interestingly, regulatory ILCs (ILCregs) are found in both human and mouse to protect intestines from inflammation through inhibition of ILC1 and ILC3 activation regulated by IL-10 secretion (114).

ILCs as mediators of inflammation in various organs play a key role in tissue remodeling and repair after recruitment and activation, which are controlled by cytokines and growth factors that are selective for each ILC member. Hence, targeting the regulation of the tissue-specific response of ILCs in inflammation may be beneficial in the setting of inflammation-driven cancer.

## Innate Lymphoid Cells in Protumor and Antitumor Effects

The role of ILCs in cancer is ambivalent as a double-edged sword by engaging in cancer immune surveillance or enhancing the unique tumor-promoting milieu, depending on the conceptual framework. We review here the different subsets of ILCs in both antitumor and protumor activities in the diverse contexts of cancer (Table 1).

**Table 1:** ILCs in different cancer types with anti-/pro-turmeric effects.

	**Cancer types**	**Functions (anti-/pro-tumor)**	**References**
ILC1s	Melanoma	Anti-tumor: ILC1s in response to IL-12, produce IFNγ and TFNα to limit tumor growth.	(115)
	Colorectal Cancer	Anti-tumor: intraepithelial CD127^−^ILC1s (ieILC1) produce cytotoxic granules to repress tumor growth.	(102)
	Mammary pre-lesion	Anti-tumor: ILC1s number expansion exhibits potent cytotoxicity to limit tumor growth in response to IL-15.	(116)
	CLL	Pro-tumor: CLL cells induce ILC1s produce IFNγ and TNFα and form immunosuppressive environment.	(117)
	AML	Pro-tumor: reduce IFNγ and TNFα production.	(118)
ILC2s	Melanoma	Anti-tumor: IL-9-producing ILC2s inhibit tumor metastasis in mouse melanoma model.	(87)
	Lymphoma	Anti-tumor: IL-33 stimulates ILC2s to secrete CXCR2 ligands that bind to CXCR2-expressing tumor cells to induce tumor cell-specific apoptosis independently of adaptive immunity.	(119)
	Lung cancer	Anti-tumor: IL-33 dependent tumor-infiltrating ILC2s mobilize from lung and facilitate dendritic cells to promote adaptive T cell response. Pro-tumor: IL-33 actives ILC2s to recruit eosinophils through ICOS/ICOSL interaction.	(120) (121)
	Colorectal cancer	Pro-tumor: Increased ILC2 are associated with high risk for development of inflammation-related CRC.	(122)
	Breast cancer	Pro-tumor: IL-33/ILC2 axis facilitates tumor growth and metastasis by recruiting MDSCs to produce IL-13. Pro-tumor: ILC2s promote breast cancer growth by upregulating Tregs through ICOS/ICOSL interaction.	(123) (124)
	Bladder cancer	Pro-tumor: ILC2/IL-13 axis modulate T cell–to-MDSC balance driving an immunosuppressive TME.	(125)
	Gastric cancer	Pro-tumor: Accumulation of ILC2s and their cytokines, IL-33 and IL-4 contribute to immune-suppressive environment.	(126)
	Prostate cancer	Pro-tumor: ILC2 elevated tumor-derived PGD2 and B7H6 and activated MDSCs via IL-13 secretion to promote tumor growth.	(123)
	APL	Pro-tumor: ILC2 elevate tumor-derived PGD2 and B7H6 to active MDSCs via IL-13 thus inducing immunosuppressive effects.	(123)
ILC3s	Melanoma	Anti-tumor: IL-12 promotes NKp46^+^CD49b^−^RORγt^+^ILC3 expansion and upregulation of VCAM1 to facilitate immune leukocyte infiltration resulting in tumor suppression.	(72)
	Lung (NSCLC)	Anti-tumor: NCR^+^ILC3s produce IL-22, TNFα, IL-8 and IL-2 to activate endothelial cells forming protective tumor-associated tertiary lymphoid structures.	(127)
	Colorectal cancer	Pro-tumor: ILC3s sustain colon cancer via production of IL-22.	(128)
	Breast cancer	Pro-tumor: CCL21-mediated recruitment of ILC3 triggers CXCL13 secretion by TME stromal cells thereby enhancing tumor cell motility and promoting lymph node metastasis.	(74)
	Hepatocellular carcinoma	Pro-tumor: NCR^−^ILC3s initiate IL-17 production upon IL-23 stimulation and promote hepatocellur carcinoma development.	(129)
	Cervical carcinoma	Pro-tumor: IL-17-producing ILC3s are also associated with poor survival in early stages.	(130)

### ILC1s

ILC1s are defined by the expression of T-bet and the production of the signature cytokine IFNγ. The prototypical member of ILC1s is the NK cell. NK cells recirculate and show strong cytotoxicity, whereas ILC1s are tissue resident and only exhibit weak cytotoxicity but produce more IFNγ and GM-CSF (131) than NK cells do (26). ILC1s in response to IL-12, a potent antitumor cytokine (115), produce the effector cytokines, IFNγ and TNFα, to limit tumor growth in a melanoma mouse model (132). The antitumor properties of ILC1s also depend on IL-15. The expanded ILC1s in mouse mammary pre-cancerous lesions exhibit potent cytotoxicity to limit tumor growth in response to IL-15 (116).

However, ILC1s also show a more complex behavior and display a detrimental role in cancer. Alteration of ILC subsets composition in patients with acute myeloid leukemia (AML), compared with the controls, could change the protective function of ILC1s by increasing their frequencies and reducing IFNγ and TNFα (118). Conversely, even though IFNγ is currently used as a clinical target for treatment of malignancies, enhanced IFNγ production by ILC1s is not associated with a better immune response but shows an immunosuppressive effect in the presence of IL-12-producing chronic lymphocytic leukemia (CLL) cells (117, 124). Moreover, ILC1s are highly abundant in colorectal cancer and produce IFNγ in response to IL-15 and IL-18, thus inducing an immunosuppressive environment (102, 133). These data suggest a dark side of IFNγ in tumor-promoting effects in cancer, and further investigation might help to understand the complex mechanisms of cancer progression in different contexts.

### ILC2s

As an alarmin, IL-33 is released from nuclei to the extracellular milieu upon cell injury caused by cell stress or damage and activates ILC2s to initiate a type 2 immune response (120). IL-33-dependent tumor-infiltrating ILC2s mobilize from lung and recruit DCs to promote the adaptive T-cell response (119). ILC2 antitumor activity under IL-33 stimulation is also involved in recruiting eosinophils to limit tumor growth and in shaping chemokine profiles in the TME under IL-5 release (134). IL-33 promotes ILC2 secretion of CXCR2 ligands that bind to CXCR2-expressing tumor cells, reinforcing tumor cell-specific apoptosis independent of adaptive immunity (135). Secretion of IL-13 by ILC2s activates CD8^+^ T cells through DC recruitment (120). In addition, ILC2s are major sources of IL-9, which can decrease tumor metastasis in a mouse model of melanoma (87).

Conversely, IL-13-producing ILC2s also show protumor immunity and are associated with a negative outcome in cancer. The ILC2/IL-13 axis modulates T cell-to-MDSC balance to drive an immunosuppressive microenvironment, whereas the inhibition of ILC2/IL-13 axis improves bladder cancer treatment (125). Similarly, the IL-33/ILC2 axis also accelerates tumor growth and promotes lung and liver metastasis by recruiting MDSCs to produce IL-13 in a mouse model of breast cancer (136), acute promyelocytic leukemia (APL), and prostate cancer (123). ILC2s impair IL-33-mediated tumor suppression by antagonizing the NK cell function during tumor growth via CD73 independently of adaptive immunity in a melanoma mouse model (119). Indeed, ILC2-derived IL-33 promotes tumor formation and metastasis in breast cancer by upregulating Treg cells through inducible co-stimulator (ICOS)/ICOS ligand (ICOSL) interaction (121), whereas IL-33 also activates ILC2s to recruit eosinophils through ICOS/ICOSL interaction during lung inflammation (137). Moreover, ILC2-derived AREG expression favors lung tumor growth and enhances resistance to apoptosis (126). Increased numbers of ILC2s were found in inflamed colonic tissue of patients with ulcerative colitis, a condition associated with a high risk for development of inflammation-driven colorectal cancer (122). Clinical studies have shown that ILC2s and their cytokines (IL-33 and IL-4) accumulate in peripheral blood and contribute to immune-suppressive environment formation in gastric cancer patients (138). The mediation of resistance to apoptosis indicates a poor prognosis in a number of cancers (139, 140).

All these data indicate that the function of ILC2s and their related cytokines are cell context dependent to facilitate either protumor or antitumor activities.

### ILC3s

Same as the other ILC subsets, ILC3s are also associated with both antitumor and protumor activities. NCR^+^ILC3 concentrates in human NSCLC and associates with intratumoral lymphoid structures, which contribute to the formation of an antitumor environment and act as predictors of favorable clinical outcome (127). In a mouse model of melanoma, increased IL-12 promotes NKp46^+^CD49b^−^RORγt^+^ILC3 expansion leading to upregulation of VCAM1 to facilitate leukocyte infiltration and the mediation of tumor suppression (72).

Conversely, ILC3s can be associated with tumor growth under different contexts. In a mouse model of colorectal cancer, NCR^+^IL-22^+^ILC3s, previously known as NK22 cells that are phenotypically distinct from LTis, sustain colon carcinogenesis in chronic inflammation (141). IL-22-producing ILC3s are crucial for the IL-22-mediated innate immune response. Notably, other subsets of ILC3s can also produce IL-22 such as CD4^+^ILC3s, which lack NCR expression in the gut (142). NCR^−^ILC3s promote hepatocellular carcinoma (HCC) development in response to IL-23 to produce IL-17, which directly limits CD8^+^ T-cell immunity by enhancing lymphocyte apoptosis and inhibiting their proliferation (129). By using an immunodeficient Rag^−/−^ mouse model, it has been shown that NKp46^−^CD4^−^ILC3 accumulation leads to IL-17 and IL-22 production, which promotes tumor development, whereas depletion of ILCs with anti-Thy1 mAbs limits tumor growth in colon cancer (141). Similarly, RORγt^+^ILC3s are associated with an increased incidence of metastasis in human breast cancer, as it was shown that depletion of ILC3 using an anti-CD90.2 antibody reduced tumor cells metastasis to lymph nodes in a syngeneic 4T1.2 mouse model (74). IL-17-producing ILC3s are also associated with poor survival in early stages of squamous cervical carcinoma (130). Interestingly, one study showed that a previously uncharacterized ILC population, ILCregs, can produce IL-22 and exhibit low cytotoxicity with a gene expression profile that overlaps with NK cells and other ILCs showing the same immunosuppressive capacity as Treg cells in human ovarian cancer studies (39).

Nevertheless, emerging evidence indicates the balance of power of ILCs in the context of cancer, and many ILC functions appear to be regulated by mechanisms distinct from those of other innate and adaptive immune cells. Notably, many studies of ILCs in cancer are based on the Rag-deficient mouse models lacking adaptive immunity. Although these experiments were crucial to analyze the fundamental features of ILC biology, these approaches have hampered the ability to study ILCs in the context of adaptive immunity and the potential interplay between ILCs and adaptive lymphocytes. The challenge now is to understand their roles and contribution within a complete immune system. The extent to which ILCs regulate the immune response (with antitumor or protumor activities) in a multitude of contexts need to be further analyzed. This is important for the understanding of their roles in cancer and for identifying targets for use in clinical treatments. In summary, although current studies reveal an important role of ILCs during tumor development, their indistinct functions in humans are incompletely understood.

## Innate Lymphoid Cell Heterogeneity and Plasticity

**Figure 5:**
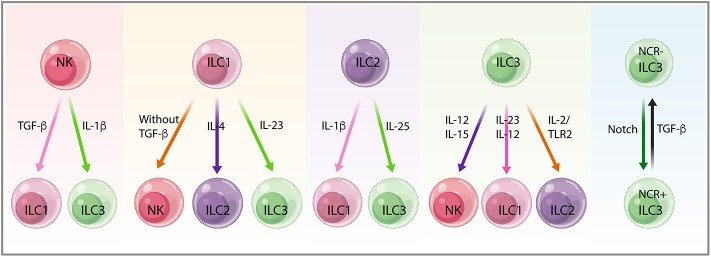
The plasticity between innate lymphoid cell (ILC) subsets. Mature ILCs display substantial plasticity depending on the distinct cytokines, the microenvironment milieus, and the diverse context of cancer. Natural killer (NK) cells convert into non-cytotoxic ILC1s upon transforming growth factor-beta (TGF-β) stimulation in the tumor microenvironment (TME) in mouse fibrosarcoma and melanoma. NK cells also convert into ILC3s in response to IL-1β, preventing stage 3 NK cells from stage 4 mature NK cells and retaining ILC3-like phenotype. ILC1s show plasticity toward all other ILCs subtypes, including NK cells, ILC2s, and ILC3s with the absence of TGF-β, the accumulation of IL-4, and under the stimulation of IL-23, respectively. Conversion of ILC2s to ILC1s needs IL-1β, whereas ILC2s in response to IL-25 differentiate into IL-17-producing ILC3s. ILC3 treatment with IL-15 and IL-12 leads to NK cell conversion, whereas plasticity from ILC3s toward ILC1s and ILC2s depends on IL-23, IL-12, and IL-2/TLR2 signals. NCR^−^ILC3 and NCR^+^ILC3 conversion needs Notch and TGF-β signals, indicating the bidirectional plasticity of ILC3s in the face of different challenges.

The pleiotropic roles of ILCs in cancer are dictated by their heterogeneity and plasticity, the noteworthy capacity of ILC subsets to convert into one another. Like helper T cells, the function and phenotype of ILCs can be modulated by extrinsic signals. In the context of cancer, mature ILCs exhibit substantial plasticity depending on the distinct cytokines present in the microenvironment milieus (143) (Figure 5).

### NK Cells to ILC1s and ILC3s

In a mouse model of fibrosarcoma and melanoma, TGF-β in the TME induced mouse NK cells to convert into non-cytotoxic ILC1s, thus weakening the control over tumor growth and metastasis (63, 144, 145). Recent studies revealed that NK cells can permanently transform to Eomes^−^T-bet-dependent ILC1-like cells independently of ongoing infection, which may explain the discrepancies of ILC1-like cells with either having antitumor function or as an inflammatory subpopulation. This exemplifies the current limitations of NK cells and ILC categorization on the basis of only their phenotypes as discrete ILC lineages (146). NK cells can also be converted into ILC3s. IL-1β stimulation prevents stage 3 NK cells from differentiating into stage 4 mature NK cells, thus keeping an ILC3-like phenotype with IL-22 production and IL-1R1 expression (147).

### ILC1s to NK Cells, ILC2s, and ILC3s

In TGF-β knock-out mice, the ILC1 number decreased in salivary glands concurrent with increased expression of CD49a, CD103, and CD69, suggesting the potential for ILC1 to NK cell plasticity (148). IL-4 accumulation leads to a shift from ILC1s toward ILC2s, which can be reversed by IL-12, in patients with severe chronic obstructive pulmonary disease (COPD) or chronic rhinosinusitis with nasal polyps, suggesting the induction of IL-12 and IL-4 signatures in ILC1s and ILC2s, respectively (149). Differentiation of ILC1s to ILC3s is driven by IL-23 (103), and this can be enhanced by IL-1β and retinoic acid produced by CD14^−^DC (103, 149, 150). The enhanced accumulation of ILC3s likely promotes tumorigenesis within different tissue locations (151).

### ILC2s to ILC1s and ILC3s

Conversion of ILC2s into ILC1s has been shown in the intestines of patients with Crohn's disease (152) and in the lungs of patients with COPD (25). Inflammation may increase ILC plasticity, thereby increasing the risk of cancer development. IL-1β also converts ILC2s into ILC1s, resulting in the increase of T-bet and IL-12Rβ2 expression, which promotes ILC1s in response to IL-12 (153). Mouse lung inflammatory ILC2s, in response to IL-25, can differentiate into IL-17-producing ILC3s that play an important role in antihelminths and antifungal immunity (154).

### ILC3s to NK Cells, ILC1s, and ILC2s

Human ILC3s treated with IL-15 and IL-12 initiate the expression of NK cell cytotoxicity markers (Eomes, CD94, CD56 NKG2A, and NKG2C), suggesting a beneficial role for ILC3-NK plasticity in cancer (155, 156). Plasticity between ILC3 and ILC1 mediated by IL-23 and also IL-12 produced by CD14^+^DC promotes polarization from ILC3s toward ILC1s, which ultimately affects tumor immunosurveillance in response to various environmental changes (2, 103, 150). IL-23-induced activation of STAT4 in NCR^+^ILC3s is a key determinant of plasticity from NCR^+^ILC3s toward ILC1s, as demonstrated by a transcriptomic analysis (157). Moreover, ILC3-to-ILC1 transition was tissue dependent and relied on transcription factor Aiolos and T-bet cooperation to repress regulatory elements active in ILC3s. This highlights the relevance of the tissue niche in creating a microenvironment that promotes ILC diversity and functional adaptation to local stimuli (158). ILC3s convert into ILC2s after activation with IL-2/TLR2 signals. In humans, IL-23 stimulation induces IL-22 secretion by ILC3s, which display clonal heterogeneity for IL-13 and IL-5 production indicating polarization *in vivo* (159).

### NCR^−^ILC3s to NCR^+^ILC3s

Mouse NCR^−^ILC3s that differentiate into NCR^+^ILC3s need Notch signals; conversely, TGF-β can convert NCR^+^ILC3s into NCR^−^ILC3s, indicating that bidirectional plasticity of ILC3s is regulated by the balance between the opposing effects of Notch and TGF-β signaling in the face of challenges (76).

Interestingly, genetic evidence shows that the differences in specific chromatin regions such as *cis*-acting enhancers and silencers that bind to transcription factors may determine the apparent plasticity of ILCs compared with the T-helper subsets. With the use of ATAC-sequencing to evaluate chromatin regions accessibility, the regulatory regions that control the distinct cytokine gene expression in ILCs exhibit poised or active status, whereas the identical regions in CD4 T helper cell counterparts become accessible only after stimulation (160, 161). These results indicate that ILC regulomes are prone to dynamic changes, and the resilient feature of ILCs directly affects their plasticity in response to the TME.

As discussed above, ILC plasticity, the ability to modify their functional phenotypes, is a fundamental phenomenon that can contribute to tumor escape mechanisms by altering the ILC-dependent tumor-surveillance system. Fully understanding the ILC plasticity control mechanisms is essential for manipulation of ILC function in the TME and designing novel therapeutic targets to prevent tumor metastasis.

## Targeting Innate Lymphoid Cells for Cancer Immunotherapy

Over the last two decades, cancer treatment has been revolutionized from targeting the tumor itself with traditional methods like surgery, chemotherapy, and radiotherapy to boosting the immune system coordinating with TIME to control cancers. Diverse approaches of immunotherapy including targeting cytokines, immune checkpoint blockade (ICB), and administration of depleting or agonistic antibodies have been used to enhance antitumor activities (27).

ILCs were first identified at the mucosal barriers, but more recent data confirm that ILCs exist in most, if not all, tissue types (75). ILCs are among the first immune cells to react to changes in environmental signaling and to shape TIME to coordinate the adaptive immune response, providing promising targets for cancer immunotherapy strategies.

### Targeting Cytokines

As immunomodulators, ILCs produce their signature cytokines and respond to various cytokines produced from other cell types and the surrounding environments. Cytokine-based immunotherapies such as IL-2 family, IL-10, IL-12, TGF-β, and several other immunosuppressive cytokines are currently under ongoing clinical trials for cancer treatment (30, 162, 163). IL-2 is a key cytokine in promoting the expansion of cytotoxic lymphocytes. IL-2 receptors, CD25, and CD122 are expressed on mouse ILC2s, whereas human ILC2s and ILC3s show high expression of CD25 only. In response to IL-2 stimulation, ILCs can be expanded and activated to facilitate antitumor effects (128, 164). However, the systemic administration of this cytokine frequently causes grade 3 and 4 adverse effects owing to its toxic profile even at the recommended doses. Several second-generation IL-2-based compounds have been engineered to reduce the high-affinity binding to IL-2Rα/CD25 and increase the half-life in circulation (163). Directed conjugation of polyethylene glycol (PEGylation) generates an inactive cytokine with a long half-life in circulation. PEGylated IL-2 (NKTR-214) has been shown to preferentially activate CD122, and this modified cytokine is currently being evaluated in phase I/II clinical trials for the treatment of various tumors (27, 165). IL-15 and IL-15 super-agonist ALT803 exhibit capabilities to increase NK cell cytotoxicity in AML patients (166) and other tumor models such as advanced solid tumors (NCT01946789), multiple myeloma (NCT02099539), relapsed hematologic malignancy (NCT01885897) (27) and metastatic NSCLC (NCT02989844) (167). IL-10 is released by innate and adaptive immune cells to regulate pro-inflammatory cytokines activity. IL-10 exhibits a context-dependent outcome in cancer, and administration of PEGylated IL-10 increases its half-life to avoid grade 3–4 immune-related adverse effects. This feature has been evaluated in a phase I clinical trial in advanced, treatment-refractory tumors (NTC02009449) (163). The antitumor and protumor effects of TGF-β in cancer are based on the control of epithelial cell growth and promoting epithelial–mesenchymal transition (EMT), respectively. Although their efficacy as monotherapy agents is limited, combined blockade of TGF-β using small molecules or antagonistic monoclonal antibodies with immune checkpoint inhibitors such as PD-1/PD-L1 (NCT02423343 and NCT02734160) shows great potential for blocking the functions of this cytokine (163). TGF-β receptor type 1 (TGFBR1) inhibitors are currently used in phase I clinical trials to treat advanced solid tumors (NCT02160106) or in combination with pomalidomide for multiple myeloma (NCT03143985) (30). Because of the intimate links between cancer and inflammation, future cancer immunotherapies may benefit from repurposing of anti-inflammatory treatments that are already in human trials for chronic inflammatory diseases such as IBD. For instance, anti-IL-12 and IL-23 blocking antibody ustekinumab has been approved for clinical trials in Crohn's disease (168). Targeting IL-17 or IFN-γ alone failed to show any effects in clinical trials, whereas targeting both provided favorable results in a preclinical model on IBD (77). Antibodies against IL-5 and anti-IL-4 receptor α subunit that block ILC2 function have provided encouraging results in patients with chronic rhinosinusitis (169). Similarly, inhibition of ILC2 function by chemoattractant receptor-homologous molecule expressed on Th2 cells (CTTH2) antagonists restores lung function in patients with asthma (170).

Cytokines are complex immune mediators with great potential in clinical cancer immunotherapy. The safety and effective use of cytokine-based drugs require a thorough knowledge of cytokine biology and advanced biotechnology in order to exploit their antitumor activity while minimizing their toxicity. In a combination with other immunomodulatory drugs such as immune checkpoint inhibitors and chimeric antigen receptor T cells, cytokine immunotherapy might become a more effective strategy in cancer treatment.

### Immune Checkpoint Blockade

ILCs share some activating and inhibitory receptors with NK and T cells, suggesting potential targets for immunotherapeutic applications. ICB is an emerging antibody-based immunotherapy providing promising strategies for cancer treatment. Antibodies targeting immune checkpoints including CTLA-4, PD-1, and PD-L1 have now been approved and used in the clinics for diverse cancer treatments (171, 172). Activation of mouse ILCs shows upregulation of PD-1, whereas increased PD-1 expression on ILC2s and ILC3 has been found in human gastrointestinal tumors, and highly expressed PD-1 and CTLA-4 were shown in human breast cancer (133, 173). Strikingly, only a minority of treated patients have benefited from of PD-1 blockade, with some others displaying drug resistance. Therefore, understanding how to shape the TME in order to increase the sensitivity to PD-1 blockade becomes indispensable. The preclinical and clinical studies in TNBC have shown that low-dose chemotherapy with doxorubicin or cisplatin or irradiation induces a favorable TME that increases the sensitivity to nivolumab treatment for PD-1 blockade to stimulate an anticancer immune response (174).

Another immune checkpoint activation protein, inducible co-stimulator (ICOS) expressed on CD4^+^T cells as well as on ILC2s and ILC3s, was identified as a crucial player in CTLA-4 blockade antitumor effects. Engagement of the ICOS pathway markedly enhances the efficacy of CTLA-4 blockade in cancer immunotherapy in a mouse model of melanoma and prostate cancer (175). Currently, an anti-ICOS agonist antibody (GSK3359609) is being used in a phase I clinical trial aimed at patients with solid tumors. In addition, therapeutic antibodies against other immune checkpoints have also been developed, such as TIM-3, LAG-3 (expressed on ILC1s only), and TIGIT (expressed on both ILC1 and ILC3s) (176). Moreover, monalizumab, an antibody against NK cell checkpoint protein NKG2A, showed the capability to enhance antitumor immunity by promoting both NK and CD8^+^ T-cell function. Based on these findings, monalizumab in combination with cetuximab is being used in head and neck squamous cell carcinoma phase II clinical trial (177).

### Depleting or Agonistic Antibody Application and Others

Both experimental mouse models and human samples showed that therapeutic targeting of ILC3 could be beneficial for autoimmune diseases. For example, depletion of ILCs by anti-Thy1 antibody improved the treatment for *Helicobacter hepaticus*-induced colitis (77). In the context of nonsolid tumors, human ILCs can be depleted before hematopoietic stem cell transplantation to treat acute leukemia (178).

Adenosine signaling belongs to a metabolic pathway that plays an important role in the immunosuppressive microenvironment favorable for cancer development. ILC1s express CD39 and CD73, activating the adenosine pathway in mouse salivary glands and increasing an immunosuppressive TIME for tumor growth, suggesting a potential target for therapeutic approaches (148).

So far, comprehensive information on the phenotypic and functional characterization of ILCs has been gathered. After the initial observation of the antitumor cytotoxicity of NK cells, other ILCs have become attractive targets for immunotherapy based on their abilities to rapidly sense and respond to signaling changes in the TME. The current challenge in cancer biology is to harness the cytokines, growth factors, and chemokines that regulate the specific function of ILCs within the TME, and further investigations will help to develop the exploitable targets for clinical applications.

## Conclusions and Perspectives

Despite advances in immunotherapies for cancer treatment, some patients still encounter few or no clinical benefits with the same treatments. Given the unique, non-redundant roles of ILCs within cancers, development of rational therapies that specifically target ILCs is likely to boost the immunotherapeutic effects in a larger group of cancer patients.

State-of-the-art technologies like single-cell RNA-sequencing (173) and high-dimensional mass spectrometry (CyTOF) analysis (179) have allowed us to investigate ILCPs, delineate distinct ILCs subsets and their developmental pathways, and identify novel mediators of antitumor immunity at single-cell resolution levels. These studies present new perspectives for exploring the complexity of ILCs in the diverse context of cancers and allow effective manipulation of ILC immunosurveillance to achieve optimal therapeutic opportunities.

## Author Contributions

ZA and TN conceived the manuscript. ZA reviewed the literature and wrote the manuscript. FF-B, SI, JD, and TN revised the manuscript.

### Conflict of Interest

The authors declare that the research was conducted in the absence of any commercial or financial relationships that could be construed as a potential conflict of interest.
